# Water Calorimetry: A Correction to the Heat Defect Calculations

**DOI:** 10.6028/jres.107.015

**Published:** 2002-04-01

**Authors:** Norman V. Klassen, Carl K. Ross

**Affiliations:** Ionizing Radiation Standards, Institute for National Measurement Standards, National Research Council, Ottawa K1A OR6 Canada

**Keywords:** dose rate, heat defect, radiation chemistry, water calorimetry

## Abstract

In a recent publication, we used a reaction model (model III) to calculate the heat defect for the irradiation of aqueous solutions with ionizing radiation at 21 °C. Subsequent work has revealed that the literature value used for one of the rate constants in the model was incorrect. A revised model (model IIIR) incorporates the correct rate constant for 21 °C. Versions of models III and IIIR were created for irradiations at 4 °C. For our current water calorimetry protocol, the values of the heat defect for H_2_/O_2_-water (water saturated with a flow of 43 % H_2_ and 57 % O_2_, by volume) at 21 °C predicted by model III and model IIIR are similar but the value for 4 °C predicted by III is 30 % smaller than the value predicted by IIIR. Model IIIR predicts that the values of the heat defect at 21 °C and 4 °C lie within the range −0.023±0.002, in agreement with the values obtained from our water calorimetry measurements done using pure water and H_2_-saturated water at 21 °C and 4 °C. The yields of hydrogen peroxide in H_2_/O_2_-water at 21 °C and 4 °C were measured and agree with the predictions of model IIIR. Our water calorimetry measurements made with pure water and H_2_-saturated water are now of sufficient quality that they can be used to determine the heat defect for H_2_/O_2_-water better than can be done by simulations. However, consistency between the three systems continues to be an excellent check on water purity which is crucial, especially for the pure water system.

## 1. Introduction

Using a water calorimeter, the absorbed dose to water from low LET (linear energy transfer) ionizing radiation such as high energy x rays and ^60^Co γ rays is obtained by measuring the temperature rise produced in the water by the absorbed dose and correcting for the effect of other materials (walls, etc.) on the temperature rise. This corrected temperature rise may be greater or lower than that which corresponds exactly to the absorbed dose because the chemical changes in the irradiated solution may be exothermic or endothermic and must be accounted for by a correction factor called the heat defect (κ_HD_),

$$\kappa_{\mathrm{HD}}=\left(E_{a}-E_{h}\right)/E_{a},$$

where *E*_a_ is the energy absorbed by the water and *E*_h_ is the energy which appears as heat.

If pure water (water saturated with nitrogen or argon), or H_2_-water (water saturated with H_2_), is used in the calorimeter, the temperature rise, after a small priming dose, will correspond exactly to the energy deposited by the absorbed dose and the heat defect will equal zero [1]. However, small amounts of oxygen or organic impurities can result in a significant heat defect. H_2_-water is less sensitive than pure water to most organic impurities. H_2_/O_2_-water (water saturated with a gas mixture of 43 % H_2_ and 57 % O_2_, by volume) is quite insensitive to organic impurities but H_2_/O_2_-water has a significant heat defect of about −0.02 which must be calculated but which is fairly constant for the first few hundred Gy and is insensitive to small changes in the ratio of H_2_ to O_2_ in the gas stream used to saturate the water.

In our previous publication, heat defects were calculated by a computer simulation which used model III to describe the radiation chemistry [1]. If simulations predict a heat defect for H_2_/O_2_-water, relative to the predictions for pure water and H_2_-water, which agree with the calorimetric measurements, we can assume with reasonable certainty that (a) there were no significant impurities in the pure water or the H_2_-water and (b) the heat defect was calculated correctly for the H_2_/O_2_-water. Since the publication of model III for 21 °C, a version of model III was created which conforms to the radiation chemistry at 4 °C, at which temperature it predicts heat defects of −0.016 for H_2_/O_2_-water and zero for pure water and H_2_-water. This is contrary to our water calorimetry measurements at 4 °C which indicate that H_2_/O_2_-water has a heat defect of −0.023 if a value of zero is assumed for pure water and H_2_-water. This disagreement led to finding an incorrect rate constant in the literature used to create model III. The correct rate constant is given here and the revised version of model III is called model IIIR. Although the error caused by using model III at 21 °C was minor, it is possible that model III could lead to significant errors at any temperature, depending on the irradiation protocol.

The predictions of the previous publication [1] were recalculated using model IIIR and compared to calculations using model III and an earlier model, model II [2]. Many water calorimeters are operated at 4 °C. The 4 °C version of model IIIR is presented. The yields of H_2_O_2_ in H_2_/O_2_-water were measured at both 4 °C and 21 °C and compared to the predictions of model IIIR. Different software is now being used to do the simulations. Results using the present and previous software are presented.

## 2. Experimental

The materials and experimental procedures have been described [1]. The water used in this study was purified by passage through a Millipore RO10 reverse osmosis unit followed by a Millipore Milli-Q UV system.^1^ Ultrapure grade N_2_, O_2_, and H_2_ were used for bubbling the solutions. Gas flowrates were measured using a Matheson model 8141 mass flowmeter. Water, saturated with N_2_ to remove air, is referred to as “pure water” because the dissolved N_2_ plays no significant role in the radiolysis.

In order to measure the H_2_O_2_ (hydrogen peroxide) produced when H_2_/O_2_ water was irradiated, 6.0 mL of the solution was irradiated in a Pyrex irradiation tube of 20 mm o.d. A silicone rubber seal at the top of the tube formed a leak-proof seal through which were inserted two concentric Pyrex tubes, sealed together, and constructed in such a way so as to permit gas to be bubbled through the 6.0 mL of water and then to exit the vessel. The solution was bubbled for 25 min to ensure saturation and then the tube assembly was raised above the water level without stopping the gas flow or causing any leak at the silicone seal. A valve between the mass flowmeter and the irradiation vessel was then partially opened to allow some of the gas to escape before reaching the vessel, thereby reducing the flow of gas across the surface of the water (to reduce evaporation) without changing the relative flowrates. This flow was maintained until the end of the irradiation. The irradiation tube was reproducibly positioned inside a Lucite tube of 37 mm o.d. and 30 mm i.d. through which water was pumped by a Neslab model RTE-111 constant temperature circulating water bath (Neslab Instruments Inc., Portsmouth, NH, USA) to control the temperature of the irradiated solution to ±0.05 °C. The solutions were irradiated with ^60^Co γ rays from an Eldorado 6 therapy unit (Atomic Energy of Canada) at a dose rate of about 2.2 Gy min^−1^. Calibration of the dose rate was done by irradiating Fricke dosimeter solutions [1, 3] in the same setup. Measurement of the H_2_O_2_ was done using the potassium iodide method [4].

## 3. Results

We have published two reaction models for calculating the heat defect for aqueous solutions used in water calorimeters. Model II was published in 1991 [2] and an “improved” model, model III, in 1997 [1]. All computer simulations followed the measurement protocol in use at the time, i.e., the same dose rate, irradiation duration and interval between irradiations. The current protocol is a set of 10 irradiation periods, of 120 s each, at a dose rate of 1.54 Gy min^−1^, each irradiation period after the first one beginning 600 s after the start of the previous one. For both measurements and simulations, the linear regressions of the temperature readings from120 s to 20 s before the start of each irradiation, and from 20 s to 120 s after the end of each irradiation, were extrapolated to the time of mid-irradiation. In the measurements, the difference between the extrapolated values at mid-irradiation represents the temperature rise caused by the absorbed dose as well as the effect of the heat defect [5]. The simulations predict the chemical changes throughout the run and the temperature changes due to these chemical changes are calculated and extrapolated to mid-irradiation. The simulations include slow chemical changes, initiated by previous irradiations, but which are still occurring during later irradiations.

We now use FACSIMILE version H012 (AEA Technology, U.K.) to run the simulations. Previously, we used MACKSIM (Atomic Energy of Canada). MACKSIM computes the chemical changes due to radiolysis and we used these changes to manually calculate the heat defect. The MACKSIM output contains the rounded-off values of the simulation and, if the change in the concentration of a species was small compared to its initial concentration, approximations had to be made to the output values in order to calculate the heat defect. FACSIMILE computes both the heat defect and the chemical changes using its full precision. The heat defects calculated by FACSIMILE and MACKSIM for common irradiation conditions at 4 °C and 21 °C never differed by more than 0.2 %.

Recently, the decision was made to operate the NRC “sealed” water calorimeter at 4 °C in order to avoid the convective heat transfer that occurs at 21 °C [6]. The heat defect had to be calculated for H_2_/O_2_-water irradiated at 4 °C. To do this, model III was adjusted to conform to 4 °C using the temperature dependencies of the *G*-values^2^ and rate constants given by Elliot [7]. The concentration of O_2_ in water saturated at 101.325 kPa is 1.40×10^−3^ mol L^−1^ at 21 °C and 1.90×10^−3^ mol L^−1^at 4 °C [8]. The concentration of H_2_ in water saturated at 101.325 kPa is 8.50×10^−4^ mol L^−1^ at 21 °C and 9.30×10^−4^ mol L^−1^ at 4 °C [9]. The pH of pure water is 7.07 at 21 °C and 7.39 at 4 °C [10]. The density of water was taken as 0.998 g cm^−3^ at 21 °C and 1.000 at 4 °C [11]. Account was taken of the fact that the calorimeter was saturated with gases at room temperature and cooled to 4 °C after sealing off the calorimeter. Simulations using model III, done for our current irradiation protocol, predicted a heat defect for H_2_/O_2_-water of −0.023 at 21 °C and −0.016 at 4 °C. However, the water calorimetry of pure water and H_2_-water, assuming a zero heat defect, consistently indicated that the heat defect for H_2_/O_2_-water should be −0.023 at both 21 °C and 4 °C [12]. The fact that the experimental results were unchanged for many refills of pure water and H_2_-water was a strong indication that the solutions were adequately pure. This led us to conclude that the heat defect predicted for H_2_/O_2_-water at 4 °C using model III was incorrect. Extensive testing revealed that the problem stemmed from the rate constants in the equilibrium defined by reactions 37 and 38 of model III [1]. To avoid confusion, we shall retain the same reaction numbers as in our earlier publication [1].

$${\mathrm{HO}}_{2}+{\mathrm{OH}}^{-}\to O_{2}^{-}+H_{2}O$$ (37)

$$O_{2}^{-}+H_{2}O\to {\mathrm{HO}}_{2}+{\mathrm{OH}}^{-}$$ (38)

The rate constants for these reactions in pure water have not been measured. Elliot [7] concluded that *k*_37_, the rate constant for reaction (37), was likely to be similar to *k*_33_.

$$\mathrm{OH}+{\mathrm{OH}}^{-}\to O^{-}+H_{2}O$$ (33)

Taking *k*_37_ = *k*_33_, Elliot evaluated *k*_38_ using the established values of equilibrium constants [7]. Due to the inadvertent replacement of the value of one equilibrium constant with that of another, Elliot calculated *k*_38_ = 1.36×10^6^ L mol^−1^ s^−1^ at 21 °C. The correct calculation yields *k*_38_ = 1.44×10^−1^ L mol^−1^ s^−1^ at 21 °C and 1.94×10^−2^ L mol^−1^ s^−1^ at 4 °C. Although never measured in water, the rate constant *k*_38_ has been measured in dimethyl formamide and in acetonitrile for solutions which contained up to 0.6 mol L^−1^ H_2_O [13,14]. The values reported for *k*_38_ in these solvents ranged from 0.5×10^−3^ L mol^−1^ s^−1^ to 3.5×10^−3^ L mol^−1^ s^−1^ with an indication in one report [14] that the value might increase with increase in water concentration. The corrected values of *k*_37_ and *k*_38_ ensure that, in neutral solutions, the equilibrium is greatly in favor of reaction (37) with the result that there is no rapid conversion of $O_{2}^{-}$ into HO_2_ during the irradiation. For this reason, the difference between the values we now accept for *k*_38_ and the smaller, published values for dimethyl formamide and acetonitrile solutions containing some water, does not affect the predicted heat defects.

We created model IIIR for 21 °C which is identical to model III [1] except that it contains the recalculated value of *k*_38_. In view of the fact that water calorimetry is often carried out at 4 °C, it is essential to have a 4 °C version of Model IIIR. This was created using Elliot’s values for the temperature dependencies of the rate constants and *G*-values [7]. The reactions and rate constants for the 4 °C version of IIIR are given in Table 1 and the *G*-values are given in Table 2. The enthalpies of formation were published earlier [2]. For H_2_/O_2_-water, the concentration of O_2_ was taken to be 8.1×10^−4^ mol L^−1^ at 21 °C and 8.2×10^−4^ mol L^−1^ at 4 °C and the concentration of H_2_ was taken to be 3.5×10^−4^ mol L^−1^ at 21 °C and 3.4×10^−4^ mol L^−1^ at 4 °C. These concentrations are a function of several factors, the gas space volume at both temperatures, the water volume at both temperatures, the solubility of the gases at both temperatures and the fact that the water was close to room temperature when it was saturated with gas.

**Fig. 1:**
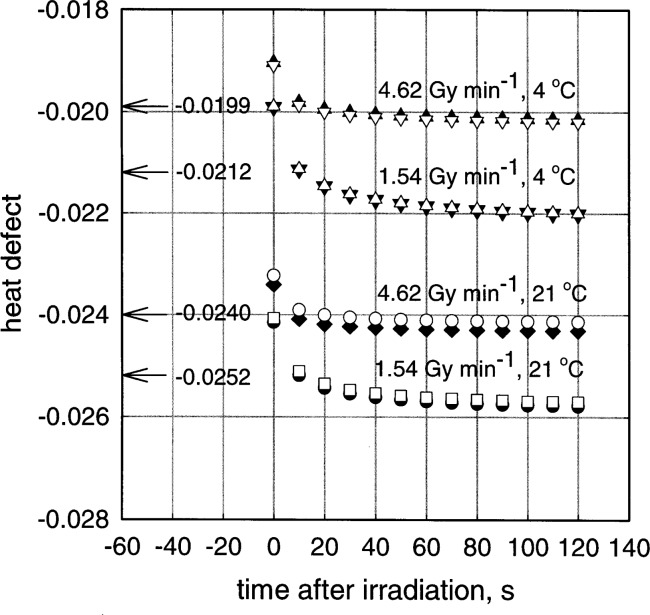
Values of the heat defect for 43/57 H_2_/O_2_ water versus time, where time = 0 s indicates the end of a 120 s irradiation and time = −60 s indicates the middle of the irradiation period. Simulations were carried out for an irradiation set of 10 consecutive irradiation periods. From the second irradiation on, each irradiation started 600 s after the start of the previous irradiation. Results for the first and tenth irradiations are shown. For the first irradiation in a set: □ represents 1.54 Gy min^−1^ at 21 °C; ○ represents 4.62 Gy min^−1^ at 21 °C; Δ represents 1.54 Gy min^−1^ at 4 °C; ∇ represents 4.62 Gy min^−1^ at 4 °C. For the tenth irradiation in a set: ● represents 1.54 Gy min^−1^ at 21 °C; ◆ represents 4.62 Gy min^−1^ at 21 °C; ▼ represents 1.54 Gy min^−1^ at 4 °C; ▲ represents 4.62 Gy min^−1^ at 4 °C. A linear regression of the values of the heat defects from 20 s to 120 s, for the first irradiation in a set was extrapolated to −60 s, i.e., the time of mid-irradiation. The value at mid-irradiation is indicated by the number and arrow at the left side of the Figure.

Figure 1 shows the heat defects for H_2_/O_2_-water versus time, at 4 °C and 21 °C, predicted by model IIIR for 2 different dose rates, 1.54 Gy min^−1^ and 4.62 Gy min^−1^. For our current irradiation protocol, the predicted heat defects are similar whether reactions (37) and (38) are included or not, which is why simulations using model II [2] did not show the same inconsistency at 4 °C as experienced with model III. Both model III and model IIIR predict close to the same values for the chemical changes at long times after the irradiation. The error in model III results in a greater slope for the linear regression used to extrapolate to mid-irradiation. This can be seen by comparing Fig. 1 in the previous publication [1] to Fig. 1 in the present paper.

Figure 1 shows that a plot of the heat defect versus time is almost identical for the 1st and the 10th irradiations of a set. The value of the heat defect, extrapolated to mid-irradiation in the same manner as the calorimetry protocol, is the value which is applied to the experimental results. The extrapolated value of the 1st run of each set is indicated to the left of the data points in Fig. 1. It can be seen that the heat defect does depend significantly on the dose rate. For our current protocol, the heat defect is predicted to be −0.0252 at 21 °C and −0.0212 at 4 °C. Careful measurements could probably determine if this predicted difference is real, i.e., whether the conversion of model IIIR from 21 °C to 4 °C is reliable.

The relationship between the *G*-values in the model and the predicted heat defect is complicated by the fact that H_2_O_2_ is produced from three different molecules H_2_O, H_2_, and O_2_, by processes, some of which are exothermic and some of which are endothermic. In order to get some idea of the dependence of the heat defect on the *G*-values chosen for the model, we calculated the heat defect using model IIIR for 4 °C using both the *G*-values in Table 2 and the *G*-values assigned to 21 °C [1]. The heat defect predicted for 4 °C using the *G*-values in Table 2 was −0.021 (see Fig. 1) compared to a value of −0.023 when the *G*-values for 21 °C were used.

We may ask whether the *G*-values, and their temperature dependencies are sufficiently well known that the difference predicted between the heat defect at 4 °C and at 21 °C is meaningful. The *G*-values for model IIIR at 4 °C are about 2 % different than the values at 21 °C. Careful measurements of *G*-values have uncertainties of 2 % to 3 % [15]. However, the conditions under which the *G*-value of one species is measured are not the same as the conditions for the measurement of the *G*-value of another species. Because of this, Elliot [7] found the measured value of *G*(H_2_O_2_) to be 6 % greater than the value that gave a material balance. The material balance which is required is that the total number of hydrogen atoms and oxygen atoms in the system must not change. We were able to achieve a predicted heat defect of −0.023 at both 21 °C and 4 °C by changing the individual *G*-values at both temperatures in an arbitrary way, while maintaining a material balance, and ensuring that none of the *G*-values was changed by more than 4 %. However, when they are created in this way, there is no logical connection between the set of *G*-values at 4 °C and the set at 21 °C, so this method cannot be recommended. Neither were we able to find any particular rate constants in model IIIR that could account for the difference between the predicted heat defects at 4 °C and 21 °C. The difference in the predicted heat defects as the rate constants and *G*-values were varied between the values assigned to 4 °C and 21 °C was taken to be a measure of the uncertainty in the predicted heat defects. We conclude that the precision of the *G*-values and rate constants in model IIIR is insufficient to reduce the uncertainty in our calculated heat defects for H_2_/O_2_-water at 4 °C or 21 °C to less than ±0.003. All uncertainties are quoted as 1 σ. Once pure water and H_2_-water have been given a sufficiently large dose to reach a steady state of chemical composition, the prediction that they have a heat defect within ±0.001 of zero is independent of the model used. Consequently, the uncertainty of the predicted heat defect would be about 0.001 for pure water and H_2_-water if the water in the calorimeter were as pure as the water in the model. Pure water and H_2_-water are more susceptible to trace impurities than is H_2_/O_2_-water. We believe that we can now determine the heat defect for H_2_/O_2_-water more reliably by basing it on the water calorimetry of pure water and H_2_-water than by basing it on model simulations as long as the calorimetry results for these three systems are consistent with one another over several fills of the calorimeter.

The radiolysis of H_2_/O_2_-water produces H_2_O_2_ whose concentration can be measured with high accuracy and precision [4]. We measured the yields of H_2_O_2_ in H_2_/O_2_-water at 4 °C and 21 °C. In these measurements, unlike the calorimetry measurements, the irradiated water was saturated at the temperature of the irradiation, hence, the concentrations of H_2_ and O_2_ were slightly different in the two cases. Simulations were carried out mimicking the conditions of the irradiations. Ten measurements each were made at 4 °C and 21 °C using dose rates of 2.22 Gy min^−1^ and irradiation times of 15 min, 30 min, and 60 min. The measured yields of H_2_O_2_ and the yields predicted by simulation are given in Table 3. The average ratio of the simulated H_2_O_2_ yields to the measured yields was 1.028±0.010 at 21 °C and 0.983±0.008 at 4 °C. When we retained the 4 °C rate constants but replaced the *G*-values in the 4 °C simulation with the *G*-values for 21 °C, the average ratio changed from 0.983±0.008 to 1.004±0.008. The latter values are shown in parentheses in Table 3. This suggests that the *G*-values chosen for 4 °C and 21 °C differ more than they ought to.

Figure 5 of our earlier publication [1] compared the simulation (model III) against the measurements of the H_2_O_2_ concentration versus dose for the irradiation of water that was saturated with H_2_ except that it contained 6×10^−7^ *M* O_2_. The use of model IIIR caused no significant change to this figure.

The irradiation of water containing H_2_ and a little O_2_ results in the production of H_2_O_2_ until the O_2_ is depleted. At that point, a chain reaction reduces the concentration of H_2_O_2_ to a low level [16], a process that produces heat. The dose at which the concentration of H_2_O_2_ decreases most rapidly produces heat the most rapidly and also corresponds to a minimum in the differential heat defect. Krauss and Roos [17] have reported both the simulation and measurement of the heat defect for the irradiation of water containing 7.6×10^−5^ mol L^−1^ O_2_ and 8.0×10^−4^ mol L^−1^ H_2_ at 20 °C. Our simulation using model IIIR is in good agreement with their results. However, a simulation of the same aqueous system at 4 °C, indicated that the minimum differential heat defect would occur at a 5.9 % higher dose at 4 °C than at 21 °C. If the radiolysis were simulated perfectly and the time at which the minimum differential heat defect occurred were measured, one might imagine using these values to calculate the dose rate. Several obstacles stand in the way of doing this with a precision of better than a few percent. If the dose rate is not identical throughout the vessel, the concentrations of H_2_O_2_ and O_2_ will change at different rates throughout the vessel and H_2_O_2_ and O_2_ will diffuse from regions of higher concentration to regions of lower concentration. This explanation was used by Krauss and Roos to explain a second “exothermal peak” which occurred when the irradiation was stopped after the first minimum and restarted 16 h later. Oxygen can also enter the water from the small gas bubble in the vessel and change the concentration of dissolved O_2_. The possibility exists that diffusion of O_2_ from the air through the seals can occur. The permeability of polyethylene to O_2_ is known [18]. The presence in the system of plastic which has been exposed to significant oxygen concentrations before the irradiation means that oxygen dissolved in the plastic will diffuse into the system. Krauss and Roos concluded that the effects of diffusion were negligible for the irradiation protocol they used. Two other problems remain. The precision of the simulation will limit the precision with which the dose rate can be measured in this way and, as shown in Table 3, our model predictions for H_2_O_2_ production in H_2_/O_2_-water at 21 °C and 4 °C differ from our measurements by about +3 % and −2 %, respectively. The other problem concerns the fact that the precision with which the O_2_ concentration can be measured is usually several percent. A study of the oxygen meter used by Krauss and Roos has been reported [19]. An error of 2.4 % in the measured O_2_ concentration was found for an O_2_ concentration of about 2.5×10^−^ 4 mol L^−1^ when the CellOx 325 was calibrated in accord with the manufacturer’s instructions. Careful temperature control and equilibration was required to reduce the error to less than 1 %. The difficulties increase as the O_2_ concentration goes down because the sensor reading at zero oxygen concentration must be subtracted from all readings.

Table 4 of our previous publication [1] listed the accumulated pre-doses required to bring the heat defect to within ±0.001 of unity for pure water and water containing traces of O_2_. These simulations were repeated using model IIIR and the results for 21 °C and 4 °C are given in Table 4 for dose rates of 1 Gy min^−1^ and 20 Gy min^−1^. It should be noted that these pre-doses do not remove the O_2_. Rather, the pre-dose establishes steady state concentrations of the species present and, in fact, the concentration of O_2_ is higher at the end of a pre-dose than it is initially.

## 4. Conclusions

The earliest measurements of water calorimetry [20] encountered problems of water purity. When H_2_/O_2_-water was shown to be insensitive to impurities, it was used as a standard system for which the heat defect was calculated using simulations [21]. If we accept a heat defect of zero for our pure water and H_2_-water, our experimental calorimetry forces us to accept a heat defect of −0.023 for H_2_/O_2_-water at both 21 °C and 4 °C. Simulations show that there are no simple changes to the *G*-values and rate constants which can reconcile this value of −0.023 with the values of −0.025 and −0.021 predicted for 21 °C and 4 °C, respectively, at 1.54 Gy min^−1^ by model IIIR. Previously, we placed a value of ±0.005 on the uncertainty in the predicted heat defect for H_2_/O_2_-water [2]. We have attempted, over the years, to improve the model and an uncertainty of ±0.003 on a predicted heat defect of about −0.023 now seems reasonable. Recently, it became clear that our water purity is sufficiently good that water calorimetry with pure water and H_2_-water consistently measure the same dose rate and that their response is stable, for many refills, when compared to H_2_/O_2_-water. This suggests that the uncertainty in the simulation of the heat defect of H_2_/O_2_-water need not be taken as the major source of uncertainty in the calculated dose rate. We conclude that water calorimetry using pure water and H_2_-water can now provide us with a better measure of the heat defect of H_2_/O_2_-water than do our simulations. We assign a value of zero to the heat defect of pure water and H_2_-water when the water meets our criteria for purity. In this regard, H_2_/O_2_-water still plays an important role as a test of the purity of the water. If H_2_/O_2_-water continues to be used in this role, simulations of H_2_/O_2_-water will still be required because the heat defect of H_2_/O_2_-water is sufficiently sensitive to the dose rate that one must carry out simulations for the operational dose rate.

## Figures and Tables

**Table 1:** Model IIIR: reactions and rate constants (4 °C)

Reactions^a^				Rate constants^b^
1	$e_{\text{aq}}^{-}+e_{\text{aq}}^{-}$	→	H_2_ + OH^−^ + OH^−^	3.48 × 10^9^
2	$e_{\text{aq}}^{-}+H$	→	H_2_ + OH^−^	1.73 × 10^10^
3	$e_{\text{aq}}^{-}+\mathrm{OH}$	→	OH^−^	2.38 × 10^10^
4	$e_{\text{aq}}^{-}+H_{2}O_{2}$	→	OH^−^ + OH	8.84 × 10^9^
5	$e_{\text{aq}}^{-}+O_{2}$	→	$O_{2}^{-}$	1.16 × 10^10^
6	$e_{\text{aq}}^{-}+O_{2}^{-}$	→	${\mathrm{HO}}_{2}^{-}+{\mathrm{OH}}^{-}$	8.48 × 10^9^
7	$e_{\text{aq}}^{-}+{\mathrm{HO}}_{2}$	→	${\mathrm{HO}}_{2}^{-}$	8.48 × 10^9^
8	H + H	→	H_2_	3.44 × 10^9^
9	H + OH	→	H_2_O	1.21 × 10^10^
10	H + H_2_O_2_	→	OH + H_2_O	3.18 × 10^7^
11	H + O_2_	→	HO_2_	9.58 × 10^9^
12	H + HO_2_	→	H_2_O_2_	7.24 × 10^9^
13	${H+O}_{2}^{-}$	→	${\mathrm{HO}}_{2}^{-}$	7.24 × 10^9^
14	OH + OH	→	H_2_O_2_	3.76 × 10^9^
15	OH + H_2_	→	H + H_2_O	2.40 ×10^7^
16	OH + H_2_O_2_	→	H_2_O + H_2_O	1.79 × 10^7^
17	OH + HO_2_	→	H_2_O + O_2_	9.08 × 10^9^
18	$\mathrm{OH}+O_{2}^{-}$	→	OH^−^ + O_2_	7.89 × 10^9^
19	HO_2_ + HO_2_	→	H_2_O_2_ + O_2_	3.72 × 10 ^5^
20	${\mathrm{HO}}_{2}+O_{2}^{-}$	→	H_2_O_2_ + O_2_ + OH^−^	5.84 × 10^7^
21	H_2_O	→	H^+^ + OH^−^	2.22 × 10^−6^
22	H^+^ + OH^−^	→	H_2_O	7.23 × 10^10^
23	H_2_O_2_	→	$H^{+}+{\mathrm{HO}}_{2}^{-}$	1.34 × 10^−2^
24	$H^{+}+{\mathrm{HO}}_{2}^{-}$	→	H_2_O_2_	3.13 × 10^10^
25	H_2_O_2_ + OH^−^	→	${\mathrm{HO}}_{2}^{-}+H_{2}O$	7.56 × 10^9^
26	${\mathrm{HO}}_{2}^{-}+H_{2}O$	→	H_2_O_2_ + OH^−^	5.45 × 10^5^
27	H	→	$e_{\text{aq}}^{-}+H^{+}$	8.83 ×10^−1^
28	$e_{\text{aq}}^{-}+H^{+}$	→	H	1.88 × 10^10^
29	$e_{\text{aq}}^{-}+H_{2}O$	→	H + OH^−^	5.08 × 10^0^
30	H + OH^−^	→	$e_{\text{aq}}^{-}+H_{2}O$	7.77 × 10^6^
31	OH	→	H^+^ + O^−^	1.34 × 10^−2^
32	H^+^ + O^−^	→	OH	3.13 × 10^10^
33	OH + OH^−^	→	O^−^ + H_2_O	7.56 × 10^9^
34	O^−^ + H_2_O	→	OH^−^ + OH	5.45 × 10^5^
35	HO_2_	→	$O_{2}^{-}+H^{+}$	4.21 × 10 ^5^
36	$O_{2}^{-}+H^{+}$	→	HO_2_	3.13 × 10^10^
37	HO_2_ + OH^−^	→	$O_{2}^{-}+H_{2}O$	7.91 × 10^9^
38	$O_{2}^{-}+H_{2}O$	→	HO_2_ + OH^−^	1.94 × 10^−2^
39	O^−^ + H_2_	→	H + OH^−^	7.95 × 10^7^
40	O^−^ + H_2_O_2_	→	$O_{2}^{-}+H_{2}O$	3.44 × 10^8^
41	$\mathrm{OH}+{\mathrm{HO}}_{2}^{-}$	→	OH^−^ + HO_2_	5.17 × 10^9^
42	OH + O^−^	→	${\mathrm{HO}}_{2}^{-}$	6.02 × 10^9^
43	$e_{\text{aq}}^{-}+{\mathrm{HO}}_{2}^{-}$	→	O^−^ + OH^−^	2.19 × 10^9^
44	$e_{\text{aq}}^{-}+O^{-}$	→	OH^−^ + OH^−^	1.82 × 10^10^
45	O^−^+ O_2_	→	$O_{3}^{-}$	2.63 × 10^9^
46	$O_{3}^{-}$	→	O_2_+ O^−^	6.70 × 10^2^
47	$O^{-}+{\mathrm{HO}}_{2}^{-}$	→	$O_{2}^{-}+{\mathrm{OH}}^{-}$	2.84 × 10^8^
48	$O^{-}+O_{2}^{-}$	→	OH^−^ + OH^−^ + O_2_	4.26 × 10^8^
49	HO_2_ + H_2_O_2_	→	OH + H_2_O + O_2_	2.90 × 10^−1^
50	$O_{2}^{-}+H_{2}O_{2}$	→	OH^−^ + OH + O_2_	9.30 × 10^−2^

a All reactions are second order except for reactions 21, 23, 27, 31, 35, and 46, which are first order.

b Second order rate constants are in the unit L mol^−1^ s^−1^. First order rate constants are in the unit s^−1^.

**Table 2:** Model IIIR: *G*-values of species

Species	*G*-value at 4 °C^a^ [(mol J^−1^]
H_2_	0.4487 × 10^−7^
H_2_O_2_	0.6817 × 10^−7^
$e_{\text{aq}}^{-}$	2.6666 × 10^−7^
H	0.5645 × 10^−7^
OH	2.7651 × 10^−7^
OH^−^	0.4455 × 10^−7^
H^+^	3.1121 × 10^−7^
H_2_O	−4.5740 × 10^−7^

a The number of significant figures is more than is warranted by the literature values but is needed for computer simulations in order that the number of H atoms and O atoms in the solution remain constant throughout a simulation.

**Table 3:** Simulation predictions and measurements of the production of H_2_O_2_ caused by the irradiation of water which had been saturated with a flow of 43 % H_2_ and 57 % O_2_, by volume

Temp. °C	Exptl trials	Irrad. min	Exptl (H_2_O_2_) µmol L^−1^	IIIR simul. µmol L^−1^	IIIR/exptl.
4	4	15	11.28 ± 0.08	11.05 (11.29)^a^	0.980 (1.001)^a^
4	4	30	22.13 ± 0.17	21.96 (22.42)^a^	0.992 (1.013)^a^
4	2	60	43.59 ± 0.36	42.58 (43.44)^a^	0.977 (0.997)^a^
21	4	15	11.40 ± 0.11	11.60	1.018
21	4	30	22.20 ± 0.36	23.05	1.038
21	2	60	43.40 ± 0.01	44.62	1.028

a The values in parentheses were calculated using predicted values from simulations for which the rate constants for 4 °C were used with the *G*-values for 21 °C.

**Table 4:** Dose to reach a heat defect of approximately 0.001 with pure water and with water containing traces of O_2_

O_2_ concentration	Dose at 21 °C 1 Gy min^−1^	Dose at 21 °C 20 Gy min^−1^	Dose at 4 °C 1 Gy min^−1^	Dose at 4 °C 20 Gy min^−1^
mol L^−1^	Gy	Gy	Gy	Gy
10^−7^	25	37	35	54
10^−8^	3	8	18	33
zero	2	7	3	28

## Footnotes

1 Certain commercial equipment, instruments, or materials are identified in this paper to foster understanding. Such identification does not imply recommendation or endorsement by the National Research Council of Canada nor the National Institute of Standards and Technology, nor does it imply that the materials or equipment identified are necessarily the best available for the purpose.

2 The value of *G* is defined as number of species created, or destroyed, per Joule of absorbed dose. The units of *G* are mol J^−1^.
